# Contributions of causes of death to differentials in life expectancy by internal migrant status in the Netherlands. A population register based study, 2015–2019

**DOI:** 10.1016/j.ssmph.2024.101690

**Published:** 2024-06-11

**Authors:** Maximilian Frentz-Göllnitz, Adrien Remund, Carel Harmsen, Lenny Stoeldraijer, Janine van der Toorn, Gabriele Doblhammer, Fanny Janssen

**Affiliations:** a Population Research Centre, Faculty of Spatial Sciences, University of Groningen, Groningen, The Netherlands; b Institute of Sociology and Demography, University of Rostock, Rostock, Germany; c Statistics Netherlands, The Hague, The Netherlands; d German Center for Neurodegenerative Diseases, Bonn, Germany; e Netherlands Interdisciplinary Demographic Institute - KNAW/University of Groningen, The Hague, The Netherlands

**Keywords:** Internal migration, Life expectancy, Causes of death, Decomposition analysis, Register data, The Netherlands

## Abstract

Important health differences exist in the context of international migration and residential mobility. Less is known about health differences regarding the medium-distance level of internal migration. This study examines life expectancy gaps between internal movers and stayers in the Netherlands and their underlying processes by assessing the contribution of different causes of death by age and sex. It uses individually-linked death counts and population exposures extracted from population registers, covering the native Dutch population aged 10+ from 2015 to 2019. The pooled data were disaggregated by causes-of-death group (neurodegenerative diseases, cardiovascular diseases, lifestyle-related mortality, external causes, and other causes), internal migrant status (movers and stayers, based on past 10-year residence in the 40 NUTS-3 [Nomenclature of Territorial Units for Statistics, level 3] regions), age, and sex. Comparing movers and stayers, we computed life expectancy at age 10 (e_10_), age- and cause-specific mortality risks, and applied decomposition methods to assess contributions of causes of death to e_10_ gaps. In the Netherlands in 2015–2019, e_10_ was lower for movers between NUTS-3 regions than stayers (males: 2.49 years; females: 3.51 years), due to excess mortality for movers at most ages. Movers only had a lower mortality than stayers at younger working ages (males: ages 20–44; females: ages 20–34). Mortality from neurodegenerative diseases and cardiovascular diseases were the largest contributors to the e_10_ gap, especially at ages 75+ and for females. Mortality from lifestyle-related and external causes of death contributed less, with the largest contributions for females aged 75–89 and males aged 45–69. The lower e_10_ of movers in the Netherlands is likely explained by health selection effects–in particular care-related moves as coping behaviour–rather than by causal effects through risk accumulation. Research focusing on regional or spatial heterogeneity of the mover-stayer health gap would be insightful to further understand these processes.

## Highlights

• Internal migrants had a lower life expectancy at age 10 than stayers.
• This disadvantage was linked to excess mortality from chronic disease at old age.
• Lifestyle-related and external mortality proved less important.
• Observed differences are rather explained by selection effects than causal effects.

## 1 Introduction

Global population growth, population ageing, and globalization have two major impacts on societies. First, more people are at risk of morbidity and mortality, adding to the burden for individuals and health care systems. Second, increasing numbers of people are migrating within or across country borders, resulting in spatial population imbalances between regions. These impacts are particularly pertinent when examining the relationship between internal migration and health, and its underlying processes.

It is well-documented that in Europe, many migrant groups have lower general mortality (Guillot et al., 2018; Ikram et al., 2016; Wallace & Wilson, 2019), also known as *Migrant Mortality Advantage*, but poorer self-rated health (Brydsten et al., 2019; Nielsen & Krasnik, 2010; Reus-Pons, Mulder, Kibele, & Janssen, 2018) than non-migrants in the host regions. Such health gaps can stem from mechanisms before migration, during the stay in the host region, or after return migration. In the context of international migration, there are several hypotheses to explain this, which can be classified into three groups: selection (at in- and out-migration), causation (socialisation, disruption, and adaptation), and data artefacts (Guillot et al., 2018). Selection pathways indicate that migrants are selected in terms of health or health-related characteristics compared to non-migrants. In contrast, causal pathways emphasise the role of differences in living environments and health behaviours. The final group, data artefacts, refers to biases in the data related to the coverage of deaths or coverage of the population. The relative importance of these mechanisms, however, is subject to academic debate, which is related to the context-specificity of their importance and the high demands on data that would be necessary to test all mechanisms simultaneously (Guillot et al., 2018; Wallace & Wilson, 2019).

However, much less is known about the relationship between regional migration and health. Based on the literature available, it is difficult to draw general conclusions regarding the health of regional migrants. Evidence in existing studies on health outcomes is inconsistent (Dijkstra, Kibele, Verweij, van der Lucht, & Janssen, 2015; Green et al., 2015; Westphal, 2016). Moreover, the extent to which the same mechanisms as indicated for international migration also apply to internal migration is rather unclear. Data artefacts are not helpful in exploration, as biases due to migration registration are much smaller within a country than across countries. Consequently, there are two main theoretical pathways for investigating the gap between movers and stayers at the regional level: selection and causation. However, because socialisation is probably less relevant in a context in which regional social and cultural differences between movers and stayers are much smaller, it is likely that selection and causation play different roles in the context of internal migration than in international migration.

The importance of these two underlying processes in driving health differentials between movers and stayers at the level of medium-distance internal migration is unclear. Because previous studies that used different scale-levels have revealed age and sex differences which could be linked to selective or causal processes of migration, formally examining the underlying contributions of age group, cause of death, and sex, would help us understand this better. No formal analysis has been performed to date, and there has been no study on the role played by causes of death in explaining health differences between internal movers and stayers.

We fill this gap by studying differences in life expectancy between internal movers and stayers in the Netherlands and examining the underlying processes by assessing the contribution of different causes of death at different ages by sex. As far as we know, ours is the first study to explore differences in life expectancy between movers and stayers at the medium-term and medium-distance level of internal migration (i.e. between larger regions) in Europe, using a formal decomposition approach to explain its drivers. We used individual-level demographic data from high-quality population register data, linked with causes-of-death data, and objectively measured internal migration.

## 2 Theoretical background and literature review

### 2.1 Explanations for health differentials between international migrants and non-migrants

European studies on international migrant health show that migrants often have lower mortality (Guillot et al., 2018; Ikram et al., 2016; Wallace & Wilson, 2019), also referred to as the *Migrant Mortality Advantage,* but poorer self-rated health (Brydsten et al., 2019; Nielsen & Krasnik, 2010; Reus-Pons, Mulder, Kibele, & Janssen, 2018) than do non-migrants in host regions. There are three main explanations to explain these differences: selection, causation, and data artefacts (Guillot et al., 2018). The relative importance of these mechanisms, however, is subject to academic debate, which is related to the context-specificity of their importance and the high demands on data that would be necessary to test all mechanisms simultaneously (Guillot et al., 2018; Wallace & Wilson, 2019).

#### 2.1.1 Selection

The selection hypothesis assumes that health gaps between migrants and non-migrants emerge indirectly, because migrants and return migrants are selected compared to stayers. This selection is based on health or health-related characteristics and can take place either when migrants enter or leave the host country. Selection at in-migration (the *Healthy Migrant Effect*) indicates that younger, more educated, and healthier individuals have higher migration chances, thus (recent) migrants may show more favourable health outcomes than non-migrants in host countries (Guillot et al., 2018; Holz, 2022; Wallace & Wilson, 2019) and origins countries (Wallace & Wilson, 2019). On the other hand, selection at out-migration (also known as the *Salmon Bias*) means that return migrants are negatively selected, with unhealthy migrants being more likely to return to their regions of origin, e.g. to benefit from social networks. Hence, those migrants who remain in the host countries are comparably healthier than non-migrants in the host countries, but unhealthier than non-migrants in the origin countries (Guillot et al., 2018; Wallace & Wilson, 2019).

#### 2.1.2 Causation

The causation hypothesis for health differences between migrants and non-migrants emphasises the role of differences in living environments and health behaviours during the stay in the host region. This hypothesis distinguishes among three pathways: disruption, adaptation, and socialisation. The disruption pathway describes how changes in the living environment, especially for newly arrived migrants, can influence levels of socioeconomic integration–e.g. availability of social networks (Brydsten et al., 2019; Kristiansen et al., 2016) or labour market position (Brydsten et al., 2019)–which often have a negative effect on their health (Brydsten et al., 2019; Kristiansen et al., 2016). The adaptation pathway argues that over time, migrants' health will converge to that of the host population as migrants adopt the health behaviours of natives and the importance of social networks in the origin country on migrant's health diminishes. Overall, this pathway is considered to have a positive health effect for migrants. The third socialisation pathway argues that the environment of origin earlier in life shapes migrant's health later in life, especially through health behaviours. Again, this pathway is generally seen as positive for migrant's health (Guillot et al., 2018; Wallace & Wilson, 2019).

#### 2.1.3 Data artefacts

Data artefacts are especially relevant in the context of migrant mortality and refer to biases in the data. Such biases might be generally related to the coverage of deaths (e.g. undercounting of deaths from migrants abroad) or the coverage of population (e.g. overcounting of migrants abroad). These could explain the lower mortality rates observed for migrants compared to non-migrants (Guillot et al., 2018; Kibele et al., 2008; Wallace & Wilson, 2022).

### 2.2 Previous research on regional migration and health

The relationship between regional migration and health has not been as widely studied as the relationship between international migration and health. The literature to date is inconclusive regarding overall health differences, but it has indicated some age and sex differences. Previous research often focused either on smaller scale levels (residential mobility or migration between municipalities) or between larger regions (e.g. NUTS-1 [Nomenclature of Territorial Units for Statistics, level 1^1^]), thereby neglecting the medium-distance scale level (NUTS-2 and NUTS-3). Generally, differences between existing studies are likely related to differences in their geographical and historical context, the definition of migrant status, or the health outcome studied.

Studies of overall health differences in the context of regional migration have primarily examined internal migration and yielded mixed results. For instance, migration within Great Britain is associated with poorer self-rated health (Green et al., 2015), and migration between municipalities within or outside the city of Eindhoven (the Netherlands) has been linked to worse health outcomes than staying in Eindhoven (Verheij et al., 1998). In contrast, migrants between federal states within Germany had an increased health satisfaction (Westphal, 2016), while migrants between Scotland and England showed an overall lower risk of limiting long-term illness compared to stayers in Scotland but not to those in England (Wallace & Kulu, 2014). However, migration between municipalities in the Netherlands was not related to self-rated health (Dijkstra, Kibele, Verweij, van der Lucht, & Janssen, 2015), and relocations within Germany were not clearly linked to physical or mental health (Holz, 2022).

A few previous studies have also hinted at an age differential, in which the association between internal migration and health is positive for older individuals (Mourits & Puschmann, 2023; Wallace & Kulu, 2014), but negative for younger individuals. For instance, from 1812 to 1962, migrants between municipalities in the province of Zeeland (the Netherlands) had lower mortality at ages 50+ than did stayers in Zeeland (Mourits & Puschmann, 2023). However, migration from the North to South in Sweden resulted in the highest mortality risk–among those aged 53 or less–for return movers, followed by movers; stayers had the lowest risk (Andersson & Drefahl, 2017).

Sex differences in the relationship between internal migration and health have also been observed. While one study found a stronger differential between movers and stayers in females (Andersson & Drefahl, 2017), another found a stronger differential in males (Westphal, 2016), and in yet another study there was no meaningful differential (Mourits & Puschmann, 2023). In addition, it has been shown that male return movers had worse health than male stayers, whereas female return movers had better health than female stayers (Wallace & Kulu, 2014).

### 2.3 Two possible pathways linking regional migration and health & hypotheses

#### 2.3.1 Pathways linking regional migration and health

The extent to which the same mechanisms for health gaps between migrants and non-migrants as indicated for international migration also apply to regional migration is rather unclear. Nevertheless, as data artefacts contribute less to biases in migration registration within a country than across countries, this leaves two possible theoretical pathways for the gap between movers and stayers at the regional level: selection and causation.

Yet, because socialisation is likely less relevant in a context in which regional social and cultural differences between movers and stayers are much smaller, it is probable that selection and causation have different roles in the context of internal migration compared to international migration. First, whereas internal migration may affect the mover's degree of integration, social networks in origin regions and behavioural differences between movers and stayers in destination regions will be less important than in international migration. In this study, we did not examine time since migration as an explanatory variable, and we defined internal movers as individuals who have a different region of residence than they did 10 years ago. Thus, on average, they will have spent five years in their current region, which is long enough for adaptive effects to develop, but short enough that some disruptive effects may still operate. Therefore, these two mechanisms were considered together for the purpose of this study. Second, initial and return migration are harder to distinguish conceptually and practically in the context of internal migration, because we classified movers based on their previous residential address as opposed to their region of birth. Thus, we examined selection effects as a whole instead of separating the respective weight of in- or out migration.

Based on these considerations, we adapt selective and causal pathways from the context of international migration and health to regional migration and health as follows.

The first pathway–related to selection–describes moving primarily as a health-selective life course event. At old age, this selection may be generally negative, as moving likely represents care-related coping behaviour as a response to poor health or living alone that is directed to institutional care or family/friends (Grundy, 2011). In fact, moves to institutional care are more likely for those with chronic health conditions as well as for older women compared to older men (Grundy & Jitlal, 2007). In contrast, at younger working ages, moving may indicate positive health selection, as young and healthy people are more likely to move by choice, related to work or their studies. In addition, being young and healthy is associated with moves to more affluent regions (Jongeneel-Grimen et al., 2013; Norman et al., 2005), which might in turn have a health-protective effect.

The second pathway–related to causation–proposes that the relationship between moving and health is complexly linked via accumulation of both risk and protective factors over the life course. First, an individual's background may moderate this relationship, as different life events (Morris, 2017; Verheij et al., 1998) and socioeconomic status (Dijkstra, Kibele, Verweij, van der Lucht, & Janssen, 2015; Westphal, 2016) affect the chances of moving. Second, after relocation takes place, changes in environmental conditions might act as a mediator. This can be through the general health effects of social networks (Smith & Christakis, 2008), living environments (Aretz, 2023; van den Berg et al., 2015), or health behaviours (Loef & Walach, 2012).

#### 2.3.2 Hypotheses

Based on the literature on regional migration and health discussed above, we formulate the following three hypotheses for our study. (1a) We expect that movers have a lower life expectancy than stayers, in line with other studies (Andersson & Drefahl, 2017; Green et al., 2015; Verheij et al., 1998). (1b) We assume that the life expectancy disadvantage of movers will be greater in females than males, as shown by a study on internal migration and mortality using recent data (Andersson & Drefahl, 2017). (2) We expect that the mover-stayer gap in life expectancy will be comparably strongly influenced by chronic health conditions around retirement age, a situation in which care-related moves are more likely (as indicated by Grundy and Jitlal (2007)). (3) Consequently, we expect that the selection pathway will be more important than the causal pathway in explaining these differences.

## 3 Material and methods

### 3.1 Data

#### 3.1.1 Study setting

This cross-sectional study with a retrospective component relied on individually linked mortality and exposure data for the native population aged 10 or above. Data for the years 2015–2019 were taken from the Dutch population register (Personal Records Database), accessed via Statistics Netherlands. This register was introduced in 1850 and digitized in 1994. It includes personal information on events such as birth, death, marriage, and change of citizenship or address (see Bakker et al., 2014; Prins, 2017). The accuracy of address data at both person- and address level from 2016 to 2021 was evaluated as being over 93% (Pagen et al., 2022).

We were provided with the following yearly aggregated data. First, population at risk by internal migrant status, age, and sex. Second, death counts by cause of death group and sex, as well as death counts by internal migrant status, cause of death, age, and sex. Strata-specific mortality counts that were between one and five were suppressed for reasons of data protection.

#### 3.1.2 Population register data

Demographic data included information on sex (male and female) and age (5-year groups: 10–14, …, 95+). Internal migrant status (movers and stayers) was defined by comparing NUTS-3 residence (40 Dutch COROP [Coordination Commission Regional Research Programme] regions) over a 10-year period. Movers were defined as individuals who reported a different region of residence than 10 years prior, while individuals with the same region of residence were defined as stayers.^2^ This was done for each year from 2015 to 2019, classifying individuals based on their residence 10 years earlier, i.e. from 2005 to 2009 (as of January 1st). This geographic and time granularity was chosen to focus on the medium-distance level of internal migration, ensuring that the decision to move was a conscious choice of relocation outside of the individual's *Daily Activity Space* (Mulder & Hooimeijer, 1999) and that movers had some time to adjust to the new environment without being amalgamated with lifetime migrants. It filters out long-term-moves, as well as short-term moves and residential mobility, which might be subject to different forces (Mulder & Malmberg, 2011). In order to avoid confusion with international migration, we removed all individuals who declared having a residence outside of the Netherlands at any time point. For the same reason, we limited our analysis to people aged 10 and above, thereby excluding those whose migrant status cannot be determined univocally.

#### 3.1.3 Causes-of-death data

Mortality data included individuals’ date of death or censoring time (December 31st or emigration), and cause of death according to ICD-10 (International Classification of Diseases, 10th revision). To find out about the underlying processes, particularly the importance of pathway 1 (coping) versus pathway 2 (accumulation), we created five cause of death groups, each with a sufficiently high number of deaths: neurodegenerative diseases, cardiovascular diseases, lifestyle-related mortality, external causes, and other causes. We expect neurodegenerative and cardiovascular disease mortality to be linked to the coping pathway because of their slow and less predictable evolution, whereas lifestyle-related mortality and external mortality are considered to be part of the accumulation pathway due to their relatively quick and predictable evolution.

(1) *Neurodegenerative diseases* comprised dementia and Alzheimer's disease, as well as Parkinson's disease. (2) *Cardiovascular diseases* included ischemic heart disease, cerebrovascular disease, and ‘other cardiovascular diseases’. (3) *Lifestyle-related mortality* contained the three main preventable risk factors of chronic disease, according to the World Health Organization (Mladovsky et al., 2009): Smoking-related mortality (based on lung cancer and ‘other smoking-related cancers’ as well as ‘non-infectious diseases of the respiratory system, including chronic obstructive pulmonary disease and chronic lower respiratory diseases’ (Zazueta-Borboa, Aburto, Permanyer, Zarulli, & Janssen, 2023)), alcohol-related mortality (based on the most important causes of death wholly attributable to alcohol (Semyonova et al., 2014)), and obesity-related mortality (based on diabetes mellitus and obesity-related mortality). (4) *External causes* consisted of the categories ‘external causes (except alcohol-related and intentional self-harm)’ and ‘intentional self-harm (except alcohol-related)’. Finally, we used the group (5) *Other causes* as a ‘control group’ for causes of death that were not included above and for which we had no prior reason to suspect differences between movers and stayers (see Supplementary Table 1 for a list of the ICD-10 codes used for each causes-of-death group.). The category of ‘symptoms and ill-defined conditions’ comprised 4.05% of the total deaths of our sample. Because this category is thematically irrelevant for our study, we proportionally redistributed these deaths among all other causes of death by internal migrant status, age, and sex. Although this assumption of equal distribution does not perfectly reflect reality, to the best of our knowledge no better classification probabilities have been designed for the context of our study.

#### 3.1.4 Data formatting

For each year from 2015 to 2019, we aggregated the mortality data by internal migrant status, cause of death, age, and sex, as well as individual demographic data by internal migrant status, age, and sex. These yearly datasets were pooled in order to increase the sample size and ensure the statistical power of our analysis. We obtained two matrices of mortality rates: one by sex and cause of death and one by cause of death, age, and sex.

### 3.2 Statistical analysis

To assess the difference in life expectancy between movers and stayers, we used the pooled data to compute life tables (Chiang, 1972) for each group of individuals for the 2015–2019 period. Anxcmigrantstatussex was taken from the national population in 2015 (as found in the Human Mortality Database (HMD, 2023)). It represents the average person-years lived in the interval between age x and x+n by those dying from cause of death c (all-cause and cause-specific) in the interval, separately by internal migrant status (total, movers, and stayers) and sex (total, male, and female). For the last age group we assumed A∞95cmigrantstatussex=1M∞95cmigrantstatussex, where M∞95cmigrantstatussex^.^indicates the strata-specific death rates. Age-specific mortality counts for suppressed cells were imputed by calculating the difference between the total number of deaths by sex and cause of death as well as the sum of deaths from non-suppressed causes by age, which was then equally distributed among the suppressed cells (by internal migrant status, cause of death, and age).

To assess the contribution of each causes-of-death group to the life expectancy gap between movers and stayers, and how this varies by age and sex, we first assessed the age- and cause-specific mortality differences between movers and stayers. We calculated all-cause and cause-specific crude death rates CDRnxcmigrantstatussex (see Preston et al., 2001) for movers and stayers (total and by sex), by dividing the respective deaths counts Dnxcmigrantstatussex by the population counts ${N_{n}}_{x}^{c^{migrant\;status^{sex}}}\text{:}$.

$${{CDR}_{n}}_{x}^{{c^{migrant\;status}}^{sex}}=\frac{\;{D_{n}}_{x}^{{c^{migrant\;status}}^{sex}}}{\;{N_{n}}_{x}^{c^{migrant\;status^{sex}}}}$$

To account for differences in the age composition between movers and stayers, we computed age-standardised mortality rates ${SDR}^{c^{observe{d^{migrant\;status}}^{sex}}}$ (see Preston et al., 2001). We used a direct standardization with the Dutch national population in 2015 (total and by sex) as standard from the Human Mortality Database (HMD, 2023):

$${SDR}^{{c^{observed^{migrant\;status}}}^{sex}}=\sum_{i=1}^{\infty }\;M_{i}^{c^{observed^{migrant\;status^{sex}}}}*\;C_{i}^{{standard}^{sex}}$$

where $M_{i}^{c^{observe{d^{migrant\;status}}^{sex}}}$ denotes the strata-specific mortality rates among the Dutch population observed and $C_{i}^{standard^{sex}}$ the strata-specific population proportions of the Dutch national population, and i is the age interval. We used the R package ‘epitools’ (Aragon, 2020) for the calculation of ${SDR}^{{c^{observed^{migrant\;status}}}^{sex}}$ and its 95% confidence intervals.

To obtain age- and cause-specific risk ratios MRRnxcsex, we divided CDRnxcmigrantstatussex for movers and stayers, separately by sex:

$${{MRR}_{n}}_{x}^{c^{sex}}=\frac{\;{{{CDR}_{n}}_{x}^{c^{movers}}}^{sex}}{\;{{{CDR}_{n}}_{x}^{c^{stayers}}}^{sex}}$$

To account for uncertainty due to the small size of some of the groups, we estimated the statistical significance of the differences between movers and stayers by means of confidence intervals for age- and cause-specific rates and for life expectancies that were calculated using a Monte Carlo approach (Andreev & Shkolnikov, 2010). For this, we generated 1000 random draws of strata-specific death counts distributed as Dnxcmovers,stayerssex∼Pois(λ) for each cell of the matrix, with mean of the distribution λ. We then computed the rates and life expectancies for each set of random draws as Dnxcmovers,stayerssex and extracted the 2.5 and 97.5 percentiles of these random draws, which we interpreted as 95% confidence intervals for rates and life expectancies.

In order to evaluate how the age- and cause-specific mortality differences between movers and stayers contribute to their life expectancy gap, we estimated the contributions of causes of death to the life expectancy gap between movers and stayers using a numerical stepwise replacement decomposition by age and cause (Andreev et al., 2002). This method decomposes the differences between aggregate measures (the life expectancies of movers and stayers) into age components and possible additional components (causes-of-death components). The algorithm takes the average between the replacement in each ‘direction’ (from population A to B, and from B to A). For this method, we used the R package ‘DemoDecomp’ (Riffe, 2018).

As a sensitivity analysis, these steps were replicated internally at Statistics Netherlands based on the original data (i.e. without suppressed strata-specific mortality counts). The results led to the same conclusions.

All analyses were carried out using R Statistical Software (v4.3.0.; R Core Team 2021).

## 4 Results

Of the 72,754,324 exposed Dutch people aged 10 and over in 2015–2019, 10.50 % are movers, 49.33 % are males, and 1.02 % died (Table 1). Whereas the crude death rates (ages 10+) are lower for movers compared to stayers, once controlled for differences in the age composition, age-standardised mortality (ages 10+) is higher for movers than stayers, both for all-cause mortality and for the causes-of-death groups. Similarly, life expectancy at age 10 (e_10_) is lower for movers than stayers, and this gap is larger for females (3.51 years) than males (2.49 years).

**Table 1:** Descriptive characteristics of the study population in the Netherlands (ages 10+, 40 NUTS-3 regions), 2015–2019, by internal migrant status and sex.

	Total		Males		Females	
	Movers	Stayers	Movers	Stayers	Movers	Stayers
**All causes combined**						
**Population at risk**	7,643,074	65,111,250	3,717,562	32,176,336	3,925,512	32,934,914
**Deaths**	52,169	687,202	22,758	335,481	29,412	351,721
**Crude death rate (per 1000)**	6.83	10.55	6.12	10.43	7.49	10.68
**Age-standardised mortality rate (per 1000) (CI)**	13.08 (12.96–13.20)	8.71 (8.69–8.73)	12.06 (11.90–12.23)	8.64 (8.61–8.67)	14.12 (13.95–14.30)	8.75 (8.72–8.78)
**Life expectancy at age 10 (years) (CI)**	69.42 (69.32–69.50)	72.46 (72.44–72.49)	68.35 (68.22–68.49)	70.84 (70.81–70.88)	70.47 (70.33–70.59)	73.98 (73.95–74.02)
**Neurodegenerative diseases**						
**Deaths** **Share of all-cause deaths (%)**	10,780 20.66	77,425 11.27	3299 14.50	27,794 8.28	7481 25.44	49,631 14.11
**Crude death rate (per 1000)**	1.41	1.19	0.89	0.86	1.91	1.51
**Age-standardised death rate (per 1000) (CI)**	2.65 (2.59–2.70)	0.92 (0.92–0.93)	1.85 (1.78–1.91)	0.68 (0.67–0.69)	3.46 (3.38–3.55)	1.17 (1.16–1.18)
**Cardiovascular diseases**						
**Deaths** **Share of all-cause deaths (%)**	12,716 24.37	185,040 26.93	5401 23.73	88,565 26.40	7316 24.87	96,475 27.43
**Crude death rate (per 1000)**	1.66	2.84	1.45	2.75	1.86	2.93
**Age-standardised death rate (per 1000) (CI)**	3.18 (3.12–3.24)	2.29 (2.28–2.30)	2.92 (2.84–3.01)	2.24 (2.23–2.26)	3.44 (3.36–3.53)	2.34 (2.32–2.35)
**Lifestyle-related mortality**						
**Deaths** **Share of all-cause deaths (%)**	10,535 20.19	184,625 26.87	5796 25.47	105,019 31.30	4739 16.11	79,606 22.63
**Crude death rate (per 1000)**	1.38	2.84	1.56	3.26	1.21	2.42
**Age-standardised death rate (per 1000) (CI)**	2.88 (2.82–2.93)	2.42 (2.41–2.43)	3.18 (3.10–3.27)	2.75 (2.73–2.77)	2.55 (2.48–2.63)	2.06 (2.05–2.08)
**External causes**						
**Deaths** **Share of all-cause deaths (%)**	3858 7.40	36,639 5.33	1914 8.41	18,681 5.57	1944 6.61	17,958 5.11
**Crude death rate (per 1000)**	0.50	0.56	0.51	0.58	0.50	0.55
**Age-standardised death rate (per 1000) (CI)**	0.77 (0.74–0.80)	0.48 (0.47–0.48)	0.73 (0.69–0.77)	0.51 (0.50–0.52)	0.81 (0.77–0.85)	0.45 (0.44–0.45)
**Other causes**						
**Deaths** **Share of all-cause deaths (%)**	14,280 27.37	203,474 29.61	6348 27.89	95,423 28.44	7932 26.97	108,051 30.72
**Crude death rate (per 1000)**	1.87	3.13	1.71	2.97	2.02	3.28
**Age-standardised death rate (per 1000) (CI)**	3.61 (3.54–3.67)	2.60 (2.59–2.61)	3.38 (3.29–3.47)	2.45 (2.44–2.47)	3.85 (3.76–3.95)	2.73 (2.71–2.74)

Data source: Statistics Netherlands. Note: CI = confidence interval.

**Fig. 1:**
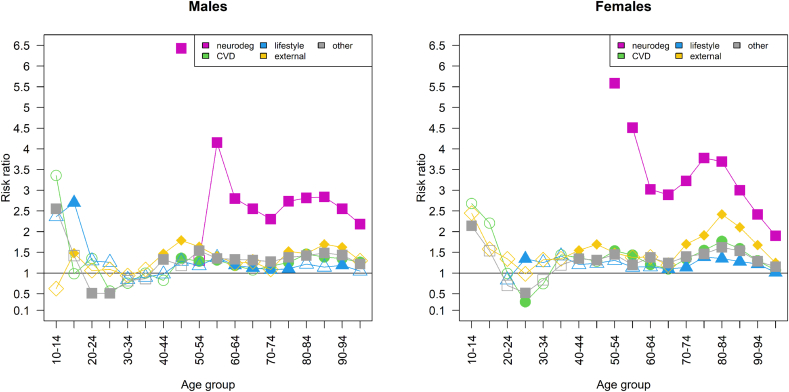
Fig. 1: Age- and cause-specific mortality risk ratios of movers and stayers (reference) in the Netherlands (ages 10+, 40 NUTS-3 regions), 2015–2019, by sex (Note for publisher: Colour is essential for understanding this figure. Please ensure that it is printed in full colour.) Data source: Statistics Netherlands. Note: neurodeg = neurodegenerative disease, CVD = cardiovascular disease. Reference = 1. Filled symbols indicate statistically significant risk ratios and blank symbols statistically non-significant risk ratios at the 95%-level. Some risk ratios could not be computed due to low numbers. The risk ratio for females' lifestyle-related mortality at age 10–14 of 10.61 is not shown. (For interpretation of the references to colour in this figure legend, the reader is referred to the Web version of this article.)

The mortality risk ratios (Fig. 1) show that for both sexes and for most age groups and causes of death, mortality risks are higher for movers than stayers. At ages 10 to 19, movers generally have excess mortality, although the differences are usually not statistically significant. This is followed by a slight dip during younger working ages (20–44 for males and 20–34 for females) where, on average, movers show lower mortality from cardiovascular disease and higher mortality from lifestyle-related and external causes of death (again, not statistically significant). After this younger working age, the risk ratios again display excess mortality for movers. A first hump in movers’ excess mortality (not statistically significant) appears at ages 45–49, followed by a slight dip around retirement age (65–69). A second hump is observed at old age (80–84), which continues with a dip in the oldest age group (95+). A comparison of the age-specific mortality rates between movers and stayers for all-cause mortality and the different causes of death (see Supplementary Fig. 1) reveals the same findings.

**Fig. 2:**
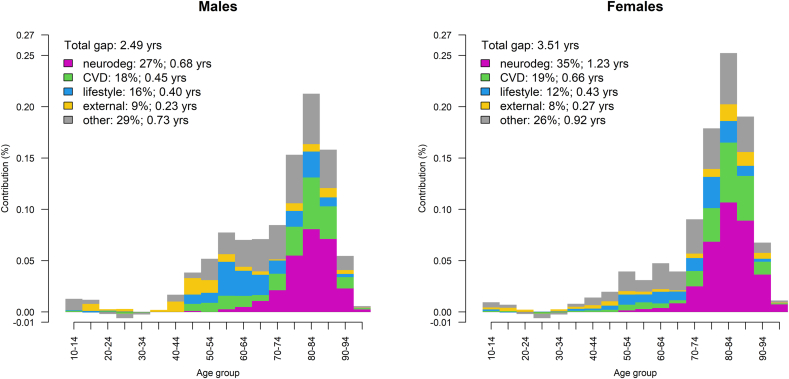
Fig. 2: Age- and cause-specific contributions to the mover disadvantage in life expectancy at age 10 (%) in the Netherlands (40 NUTS-3 regions), 2015–2019, by sex (Note for publisher: Colour is essential for understanding this figure. Please ensure that it is printed in full colour.) Data source: Statistics Netherlands. Note: yrs = years, neurodeg = neurodegenerative disease, CVD = cardiovascular disease. Positive values indicate mover disadvantage, negative values indicate mover advantage. (For interpretation of the references to colour in this figure legend, the reader is referred to the Web version of this article.)

Our decomposition analysis reveals that neurodegenerative disease mortality is the strongest driver of the e_10_ disadvantage for movers, with the highest contributions at old age (75+) and a larger impact on females (35 %) than males (27 %) (Fig. 2). Cardiovascular disease mortality explains approximately 18–19 % of the total gap for males and females, and is also most relevant at old age (75+). Lifestyle-related mortality contributes 16 % among males and 12 % among females, and is notably more important for males at ages 45–69 and females at ages 75–89. External mortality constitutes 9 and 8 % of the gap for male and female movers respectively, with a comparably stronger impact for females at ages 75–89 and for males at ages 45–69. All in all, despite strong risk ratios in adolescence and mid-life (ages 10 to 39), these ages contribute very little to the e_10_ disadvantage for movers because of the low overall mortality levels.

## 5 Discussion

### 5.1 Summary of the results

Using high-quality Dutch register data, we found that remaining life expectancy at age 10 is 2.5 years lower for males and 3.5 years lower for females who lived in a different NUTS-3 region (40 in total) within the Netherlands 10 years prior, than for their counterparts who had stayed in the same region. The mortality of movers exceeded mortality of stayers at most ages, except at younger working ages (20–44 for males, 20–34 for females). Our formal decomposition analysis showed that neurodegenerative and cardiovascular disease mortality were the largest contributors to the e_10_ gap, especially at ages 75+ and especially for females. Lifestyle-related and external mortality played a comparatively smaller role, with the largest contributions for females aged 75–89 and males aged 45–69.

### 5.2 Discussion of the results

The lower e_10_ we observed for movers compared to stayers is in line with our hypothesis 1a and consistent with some of the previous studies finding a negative association between internal migration and health (Andersson & Drefahl, 2017; Green et al., 2015; Verheij et al., 1998). Diverging findings may depend partly on the scale level or length of migration, or the sample studied. Those studies showing a positive association either assessed migration at the NUTS-1 level during a nine-year study period (Westphal, 2016), lifetime migration between states (Wallace & Kulu, 2014), or lifetime family migration between municipalities (Mourits & Puschmann, 2023). Furthermore, the studies which indicated no (clear) differences either analysed migration between municipalities during a five-year period while also including international migrants in the sample (Dijkstra, Kibele, Verweij, van der Lucht, & Janssen, 2015), or any work-related migration within 10 years prior (Holz, 2022).

Our formal decomposition analysis revealed a strong contribution of excess mortality from neurodegenerative and cardiovascular diseases at ages 75+ to the observed e_10_ mover disadvantage. Therefore, we accept hypothesis 2, which stated that chronic health conditions around retirement age are especially important in explaining the e_10_ difference. These findings indicate the importance of coping behaviours that are linked to health selection. That is, among older people, those in poor health and those unmarried are more likely to move to institutional care or family/friends (Grundy, 2011). In the Netherlands, both family/friends and health and are important motives for moves at ages 65+, while the average distance is higher for moves related to family/friends (ca. 30 km) compared to health (within 10 km) (Lennartz et al., 2023). This suggests that our results may reflect the moves of those in poor health to family/friends or specialised institutions, entailing larger distances as opposed to seeking care in the neighbourhood. Therefore, especially those regions receiving a high number of both older and female movers might have an increased demand for professional and informal carers.

Excess mortality from lifestyle-related and external causes of death played a comparably smaller role in explaining the e_10_ gap between movers and stayers, with larger contributions for males aged 45–69 and females aged 75–89. We thus accept hypothesis 3, which assumed that selection is more important than causation in explaining the e_10_ difference. The observed excess lifestyle-related mortality among movers is possibly related to risk accumulation through socioeconomic circumstances and life events. First, while moving is associated with health improvements for the highly educated, it is the opposite for those with less education (Stawarz et al., 2022). Second, positive and negative life course events can trigger moves, while experiencing negative life events makes it more likely to stay in poor environments than in good ones (Morris, 2017), which may subsequently impair health (Aretz, 2023).

Altogether this indicates a clear mortality disadvantage for movers and would mean that selection effects are more important than causal effects in explaining the e_10_ disadvantage among movers. This is in contrast with the literature on mortality of international migrants in Europe that finds that many migrant groups have lower overall mortality (Guillot et al., 2018; Ikram et al., 2016; Wallace & Wilson, 2019). Although we find evidence for selection into internal migration as a coping mechanism, this appears to be linked to poor rather than good health. Indeed, the threshold for care-related moves within a country may be lower than for care-related moves between countries. Similarly, causal effects of life-course accumulation also have a negative influence on health of internal movers, and–while this pathway was comparatively less relevant–the direction is the same when compared to international migration. While data artefacts are much less relevant in the context of internal migration, they do partially explain the lower mortality of international migrants relative to non-migrants. This suggests that different pathways are indeed causing the differentials for internal movers compared to international migrants.

We also observed a larger gap in the e_10_ disadvantage between movers and stayers for females compared to males, which is in accordance with hypothesis 1b. Such a sex difference can likely be explained by sex differences in longevity. That is, neurodegenerative disease mortality explained a larger share of the differential for females than males, which could be due to older women having a higher chance of moving to institutional care than older men (Grundy & Jitlal, 2007), because they often outlive their partners.

Our results also showed that, in contrast to all other ages, movers had slightly lower mortality than stayers at ages 20–34. This difference is probably related to health selection, meaning that among young adults, healthy individuals may have higher chances of internal migration motivated by work or studies. In addition, being young and healthy is associated with moves to more affluent regions (Jongeneel-Grimen et al., 2013; Norman et al., 2005), which might in turn have a health-protective effect. In the Dutch context, work and study are important reasons for young adults to move, while these types of migration are, on average, those with the largest moving distances (ca. 42.5 km and 45 km, respectively) (Lennartz et al., 2023).

### 5.3 Strengths and limitations

Our study has several strengths. First, we made use of high-quality register data, including individually-linked mortality and exposure data. Our results are therefore generalizable to the native-born population of the Netherlands aged 10+ in the period 2015–2019. Second, the retrospective information on place of residence allowed us to have an objective measure of internal migrant status. Third, by excluding international migrants from our study, we limited potential biases due to registration errors (data artefacts) and unobserved heterogeneity in the health of this group. Fourth, with an age-cause decomposition we used a formal approach to explain observed differences. Apparently this had not been not done in the field of regional migration and health in Europe before, although this demographic technique is widely used in public health (Aburto et al., 2018; Sauerberg, 2021).

There are also limitations to this study. First, our analysis was descriptive and based on four broad strata (internal migrant status, cause of death, sex, and age). This could hamper our assessment of underlying processes linked to omitted variables such as an individual's socioeconomic status, health behaviours, or area characteristics, which could explain the similar age patterns observed for all causes of death. For instance, we know that regional migration may contribute to health inequalities between regions by selective sorting via social class and area affluency (Darlington-Pollock & Norman, 2022). Nevertheless, because we conceptually linked the causes of death to our pathways, we were still able to assess the likely relative importance of the two hypothesised underlying processes. Second, we did not distinguish return movers from other movers in our analysis. It is thus possible that our results are influenced by individuals who move back and forth between two time points, and who were erroneously treated as stayers in (part of) our analysis. Assuming that return movers tend to be unhealthier individuals, this would have overestimated the effect of the difference between movers and stayers. However, because we used yearly data, this would have applied to only a very small group of movers and therefore likely has not significantly biased the results. Third, due to data availability, we estimated lifestyle-related mortality based purely on underlying causes-of-death information for smoking-, alcohol- and obesity-attributable mortality, compared to using an approach that also takes into account lifestyle-related mortality from causes of death that are partly related to that lifestyle. Examples for this are an indirect estimation method for smoking (Janssen, van Wissen, & Kunst, 2013), a population-attributable fraction approach for alcohol (Trias-Llimós, Martikainen, Mäkelä, & Janssen, 2018), or a multiple causes of death approach for obesity (Adair & Lopez, 2020). This has likely resulted in an underestimation of lifestyle-attributable mortality and its contribution to the e_10_ disadvantage of movers. Moreover, cardiovascular disease mortality likely includes lifestyle-related mortality as well. However, given the much larger contribution of neurodegenerative diseases compared to external causes, our conclusion that selection effects are probably more important than causal effects seems to hold. All in all, these limitations are inherent to studying mortality differentials between movers and stayers in a relatively small country, but our approach still yields meaningful outcomes.

## 6 Conclusions

This study has shown that among natives aged 10+ in the Netherlands 2015–2019, movers between NUTS-3 regions over a 10-year period have lower life expectancy and higher mortality compared to stayers. Health selection effects–in particular care-related moves as coping behaviour–appear to be more relevant in explaining these findings than causal effects through risk accumulation. Our results are different from the literature on the mortality of international migrants in Europe, suggesting that different pathways are indeed causing the health differentials at different scale levels. Exploring additional factors such as heterogeneity between regions based on geographical location or spatial characteristics will be a promising field of investigation to further understand exactly how health and internal migration status are linked.

## 7 Declarations of interest

None.

## Ethical statement

Our analysis is based on purely aggregate-level data. The access to the underlying individual-level data was handled exclusively by Statistics Netherlands. Therefore, no ethical approval was required for our study.

## Funding

FJ has received funding from the 10.13039/501100003246Netherlands Organisation for Scientific Research (NWO) (grant no. VIC.191.019).

## CRediT authorship contribution statement

**Maximilian Frentz-Göllnitz:** Writing – review & editing, Writing – original draft, Visualization, Methodology, Investigation, Formal analysis, Conceptualization. **Adrien Remund:** Writing – review & editing, Writing – original draft, Visualization, Supervision, Methodology, Investigation, Formal analysis, Conceptualization. **Carel Harmsen:** Writing – review & editing, Resources, Methodology, Investigation, Conceptualization. **Lenny Stoeldraijer:** Writing – review & editing, Resources, Methodology, Investigation. **Janine van der Toorn:** Writing – review & editing, Resources, Methodology, Investigation. **Gabriele Doblhammer:** Writing – review & editing, Methodology. **Fanny Janssen:** Writing – review & editing, Supervision, Methodology, Conceptualization.

## Data Availability

The authors do not have permission to share data.

## Footnotes

1 There are three levels of NUTS regions: NUTS-1, NUTS-2, and NUTS-3.

2 COROP regions have an average size (surface) of 841.18 km^2^ (median = 852.69 km^2^, standard deviation = 482.19 km^2^) (Statistics Netherlands, 2024). Statistics on moving distances between COROP regions are not available, but in 2002, the average moving distance for households within the Netherlands was 16.7 km (Ekamper & van Huis, 2005) and the share of persons moving across municipal borders (i.e. including across COROP regions) was one third (Feijten & Visser, 2005). Moves across COROPS are thus above this average, which suggests a change in the *Daily Activity Space*.

## References

[bib1] Aburto J.M., Wensink M., van Raalte A., Lindahl-Jacobsen R. (2018). Potential gains in life expectancy by reducing inequality of lifespans in Denmark: An international comparison and cause-of-death analysis. BMC Public Health.

[bib2] Adair T., Lopez A.D. (2020). The role of overweight and obesity in adverse cardiovascular disease mortality trends: An analysis of multiple cause of death data from Australia and the USA. BMC Medicine.

[bib3] Andersson G., Drefahl S. (2017). Long-distance migration and mortality in Sweden: Testing the Salmon Bias and healthy migrant hypotheses. Population, Space and Place.

[bib4] Andreev E.M., Shkolnikov V.M. (2010).

[bib5] Andreev E.M., Shkolnikov V.M., Begun A.Z. (2002). Algorithm for decomposition of differences between aggregate demographic measures and its application to life expectancies, healthy life expectancies, parity-progression ratios and total fertility rates. Demographic Research.

[bib11] Aragon T.J. (2020). epitools: Epidemiology Tools. R package version 0.5-10.1.

[bib12] Aretz B. (2023).

[bib13] Bakker B.F.M., van Rooijen J., van Toor L. (2014). The System of social statistical datasets of Statistics Netherlands: An integral approach to the production of register-based social statistics. Statistical Journal of the IAOS.

[bib14] Brydsten A., Rostila M., Dunlavy A. (2019). Social integration and mental health—a decomposition approach to mental health inequalities between the foreign-born and native-born in Sweden. International Journal for Equity in Health.

[bib15] Chiang C.L. (1972). On constructing current life tables. Journal of the American Statistical Association.

[bib16] Darlington-Pollock F., Norman P. (2022). Establishing a framework of analysis for selective sorting and changing health gradients. Population, Space and Place.

[bib7] Dijkstra A., Kibele E.U.B., Verweij A., van der Lucht F., Janssen F. (2015). Can selective migration explain why health is worse in regions with population decline?: A study on migration and self-rated health in the Netherlands. European Journal of Public Health.

[bib17] Ekamper P., van Huis M. (2005). Verhuizingen en huishoudensveranderingen in Nederland: Verschillen tussen COROP-regio’s. Bevolkingstrends.

[bib18] Feijten P., Visser P. (2005). Binnenlandse migratie: Verhuismotieven en verhuisafstand. Bevolkingstrends.

[bib19] Green M.A., Subramanian S.V., Vickers D., Dorling D. (2015). Internal migration, area effects and health: Does where you move to impact upon your health?. Social Science & Medicine.

[bib20] Grundy E. (2011). Household transitions and subsequent mortality among older people in England and Wales: Trends over three decades. Journal of Epidemiology & Community Health.

[bib21] Grundy E., Jitlal M. (2007). Socio-demographic variations in moves to institutional care 1991 2001: A record linkage study from England and wales. Age and Ageing.

[bib22] Guillot M., Khlat M., Elo I., Solignac M., Wallace M. (2018). Understanding age variations in the migrant mortality advantage: An international comparative perspective. PLoS One.

[bib23] HMD (2023). https://mortality.org/Home/Index.

[bib24] Holz M. (2022). Health inequalities in Germany: Differences in the ‘Healthy migrant effect’ of European, non-European and internal migrants. Journal of Ethnic and Migration Studies.

[bib25] Ikram U.Z., Mackenbach J.P., Harding S., Rey G., Bhopal R.S., Regidor E., Rosato M., Juel K., Stronks K., Kunst A.E. (2016). All-cause and cause-specific mortality of different migrant populations in Europe. European Journal of Epidemiology.

[bib6] Janssen F., van Wissen, Kunst A.E. (2013). Including the smoking epidemic in internationally coherent mortality projections. Demography.

[bib26] Jongeneel-Grimen B., Droomers M., Stronks K., van Oers J.A.M., Kunst A.E. (2013). Migration and geographical inequalities in health in The Netherlands: An investigation of age patterns. International Journal of Public Health.

[bib27] Kibele E., Scholz R., Shkolnikov V.M. (2008). Low migrant mortality in Germany for men aged 65 and older: Fact or artifact?. European Journal of Epidemiology.

[bib28] Kristiansen M., Razum O., Tezcan-Güntekin H., Krasnik A. (2016). Aging and health among migrants in a European perspective. Public Health Reviews.

[bib29] Lennartz C., Troost S., Schilder F. (2023). Een empirische analyse van het verhuisgedrag van huishoudens in Nederland.

[bib30] Loef M., Walach H. (2012). The combined effects of healthy lifestyle behaviors on all cause mortality: A systematic review and meta-analysis. Preventive Medicine.

[bib31] Mladovsky P., Allin S., Masseria C., Hernández-Quevedo C., McDaid D., Mossialos E. (2009). Health in the European union: Trends and analysis.

[bib32] Morris T. (2017). Examining the influence of major life events as drivers of residential mobility and neighbourhood transitions. Demographic Research.

[bib33] Mourits R.J., Puschmann P. (2023). Exploring familial factors in the migrant mortality advantage among domestic migrants in later life: Zeeland, The Netherlands, 1812–1962. SSM - Population Health.

[bib34] Mulder C.H., Hooimeijer P., van Wissen L.J.G., Dykstra P.A. (1999). Population issues: An interdisciplinary focus.

[bib35] Mulder C.H., Malmberg G. (2011). Moving related to separation: Who moves and to what distance. Environment & Planning A: Economy and Space.

[bib36] Nielsen S.S., Krasnik A. (2010). Poorer self-perceived health among migrants and ethnic minorities versus the majority population in Europe: A systematic review. International Journal of Public Health.

[bib37] Norman P., Boyle P., Rees P. (2005). Selective migration, health and deprivation: A longitudinal analysis. Social Science & Medicine.

[bib38] Pagen L., Bouhuijs I., Oksana B., Joosten M. (2022).

[bib39] Preston S.H., Heuveline P., Guillot M. (2001).

[bib40] Prins K. (2017). Population register data, basis for the Netherlands’ Population Statistics. Bevolkingstrends.

[bib54] Reus-Pons M., Mulder C.H., Kibele E.U.B., Janssen F. (2018). Differences in the health transition patterns of migrants and non-migrants aged 50 and older in southern and western Europe (2004-2015). BMC Medicine.

[bib41] Riffe T. (2018). DemoDecomp: Decompose demographic functions. R package version 1.0.1.

[bib42] Sauerberg M. (2021). The impact of population's educational composition on Healthy Life Years: An empirical illustration of 16 European countries. SSM - Population Health.

[bib43] Semyonova V.G., Gavrilova N.S., Sabgayda T.P., Antonova O.M., Nikitina S.Y., Evdokushkina G.N. (2014). Mortality in an international perspective.

[bib44] Smith K.P., Christakis N.A. (2008). Social networks and health. Annual Review of Sociology.

[bib45] Statistics Netherlands (2024). https://opendata.cbs.nl/statline/#/CBS/nl/dataset/70072ned/table?dl=A4084.

[bib46] Stawarz N., Arránz Becker O., Rüger H. (2022). Work-related internal migration and changes in mental and physical health: A longitudinal study using German data. Health & Place.

[bib53] Trias-Llimós S., Martikainen P., Mäkelä P., Janssen F. (2018). Comparison of different approaches for estimating age-specific alcohol-attributable mortality: The cases of France and Finland. PloS One.

[bib47] van den Berg M., Wendel-Vos W., van Poppel M., Kemper H., van Mechelen W., Maas J. (2015). Health benefits of green spaces in the living environment: A systematic review of epidemiological studies. Urban Forestry and Urban Greening.

[bib48] Verheij R.A., Mheen H.D., Bakker D. H. de, Groenewegen P.P., Mackenbach J.P. (1998). Urban-rural variations in health in The Netherlands: Does selective migration play a part?. Journal of Epidemiology & Community Health.

[bib49] Wallace M., Kulu H. (2014). Migration and health in England and Scotland: A study of migrant selectivity and Salmon Bias. Population, Space and Place.

[bib50] Wallace M., Wilson B. (2019). Migrant mortality advantage versus origin and the selection hypothesis. Population and Development Review.

[bib51] Wallace M., Wilson B. (2022). Age variations and population over-coverage: Is low mortality among migrants merely a data artefact?. Population Studies.

[bib52] Westphal C. (2016). Healthy migrants? Health selection of internal migrants in Germany. European Journal of Population.

[bib10] Zazueta-Borboa J.-D., Aburto J.M., Permanyer I., Zarulli V., Janssen F. (2023). Contributions of age groups and causes of death to the sex gap in lifespan variation in Europe. *Population Studies*.

