# Supplementary material for: Contributions of causes of death to differentials in life expectancy by internal migrant status in the Netherlands. A population register based study, 2015–2019
Source: SSM Popul Health. 2024 Jun 11;27:101690. doi: 10.1016/j.ssmph.2024.101690 (PMC11259871; doi:10.1016/j.ssmph.2024.101690)
Supplement: Multimedia component 1 [file mmc1.docx]

# Supplementary material

Supplementary Table 1: List of ICD-10 codes for causes-of-death groups

| Group | Causes of death | ICD-10 |
| --- | --- | --- |
| Neurodegenerative diseases | Dementia and Alzheimer’s disease  Parkinson’s disease | F00-F03, G30  G20 |
| Cardiovascular diseases | Ischemic heart disease | I20-I25 |
|  | Cerebrovascular disease | I60-I69 |
|  | Other cardiovascular diseases | I00-I15, I26-I42.5, I42.7-I52, I70-I99 |
| Lifestyle-related mortality | Smoking-related mortality  Lung cancer  Other smoking-related cancers  Non-infectious diseases of the respiratory system, including chronic obstructive pulmonary disease and chronic lower respiratory diseases | C33-C34  C00-C21, C25, C30-C32, C64-C68  J30-J33, J34.1-J35, J37-J38, J39.2-J84, J90-J99 |
|  | Alcohol-related mortality | F10, G31.2, G62.1, G72.1, I42.6, K29.2, K70, K86.0, Q86.0, X45, X65, Y15 |
|  | Obesity-related mortality  Diabetes mellitus  Obesity | E10-E14  E65-E66 |
| External causes | External causes (except alcohol-related and intentional self-harm)  Accidents  Assault  Other | V01-X44, X46-X59  X85-Y09  Y14, Y16-Y86, Y87.1-Y89 |
|  | Intentional self-harm (except alcohol-related) | X60-X64, X66-X84, Y87.0 |
| Symptoms and ill-defined conditions |  | R00-R99 |
| Other causes | Infectious (respiratory) diseases | A00-B99, J00-J22, J34.0, J36, J39.0, J39.1, J85-J86 |
|  | Other neoplasms | C22-C24, C26, C37-C63, C69-C97 |
|  | Residual causes | All codes not included above |

Supplementary Fig. 1: Age-specific mortality rates of movers and stayers in the Netherlands (ages 10+, 40 NUTS-3 regions), 2015-2019, all causes combined (Panel A) and by cause of death (Panels B-F), by sex

A: All-cause mortality


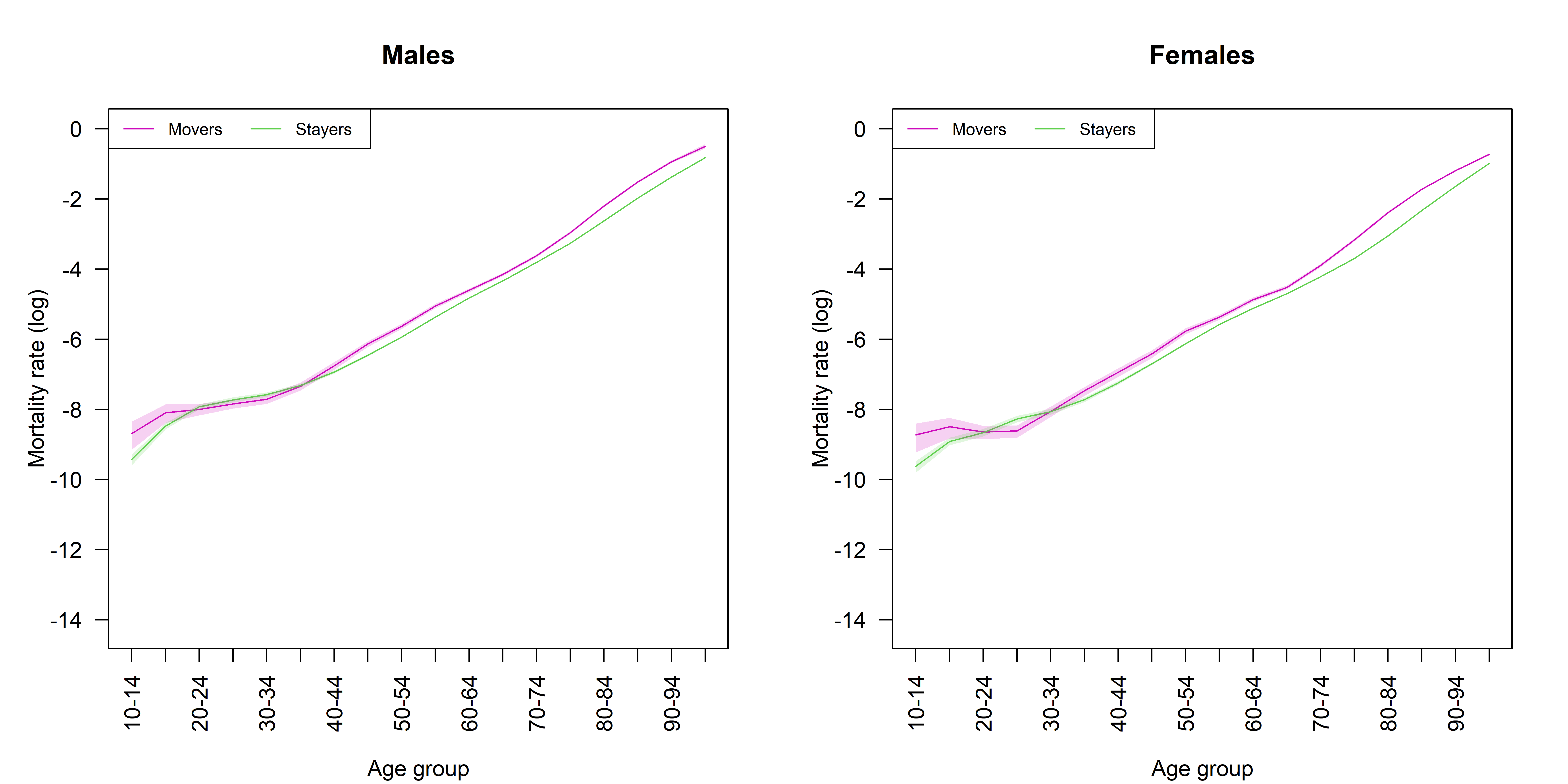


B: Neurodegenerative disease mortality


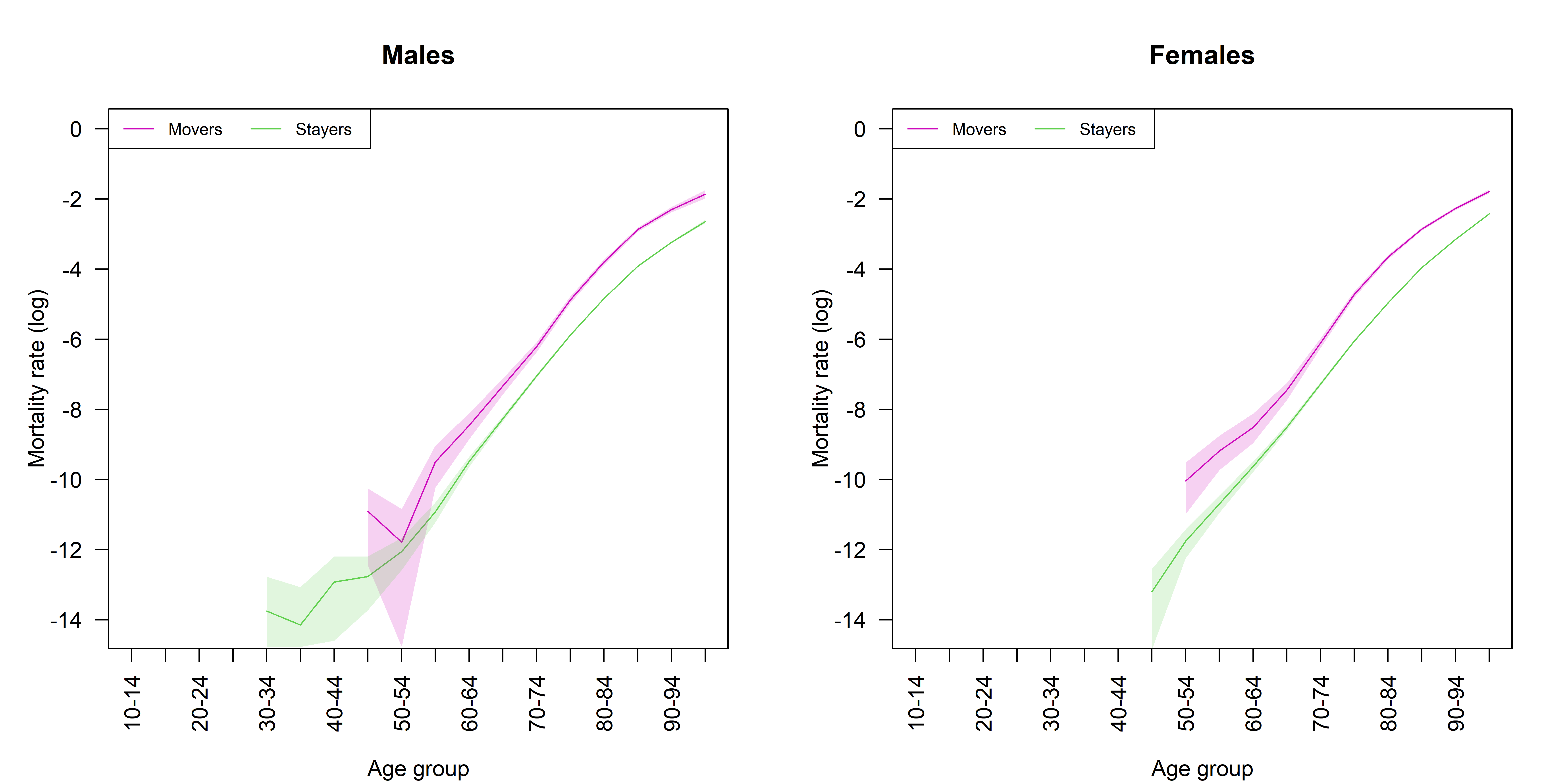


C: Cardiovascular disease mortality


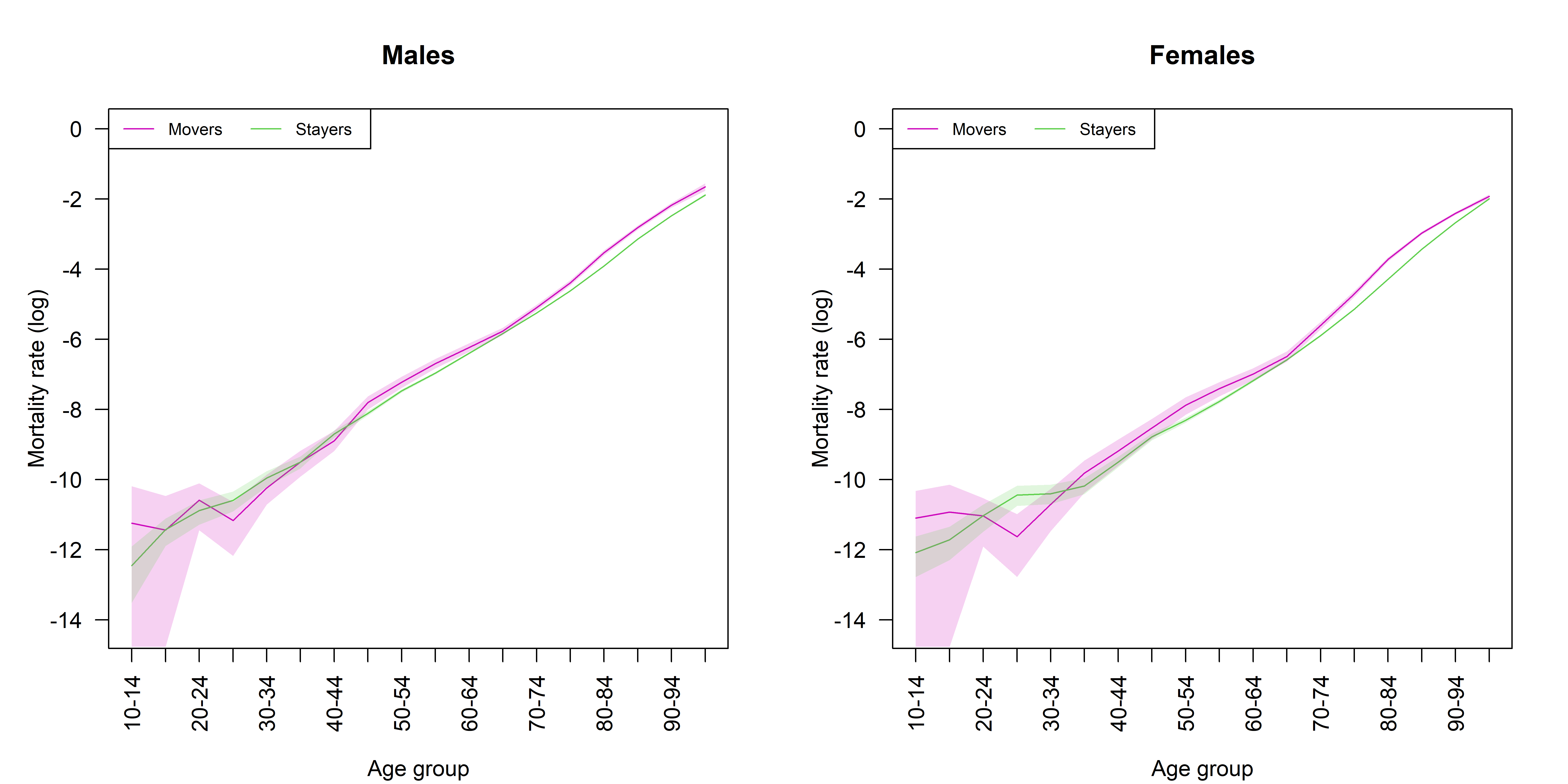


D: Lifestyle-related mortality


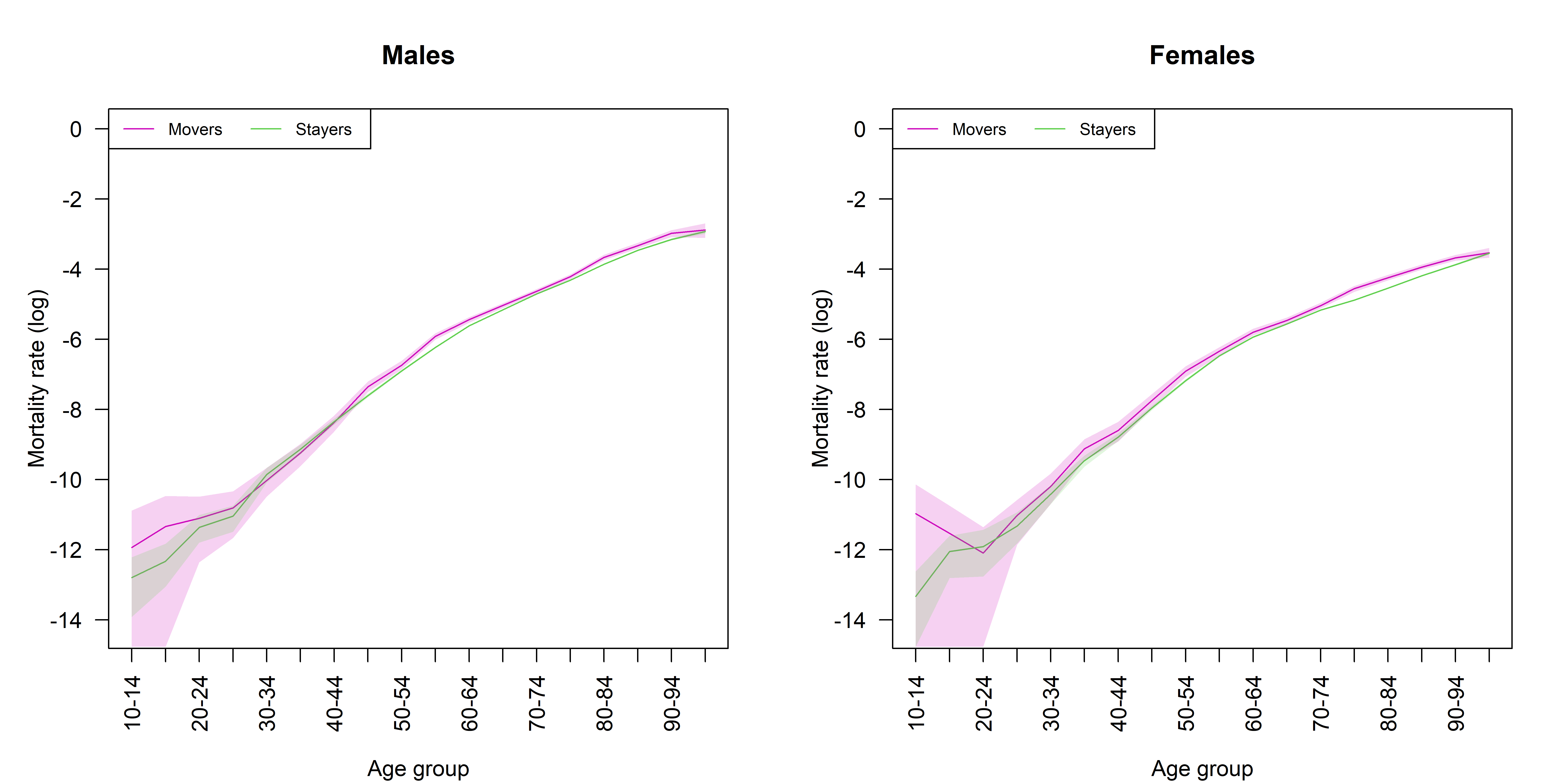


E: External mortality


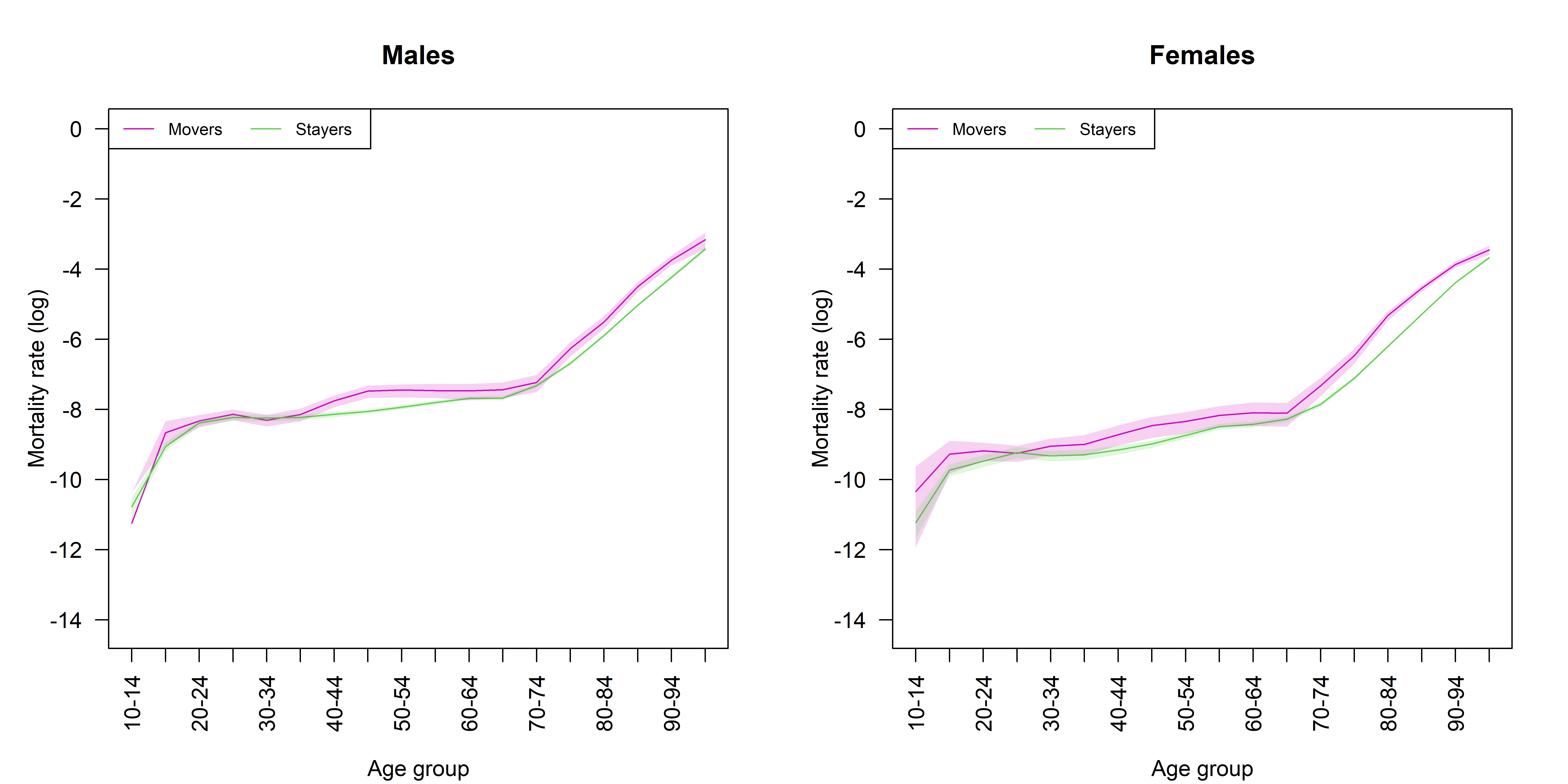


F: Other mortality


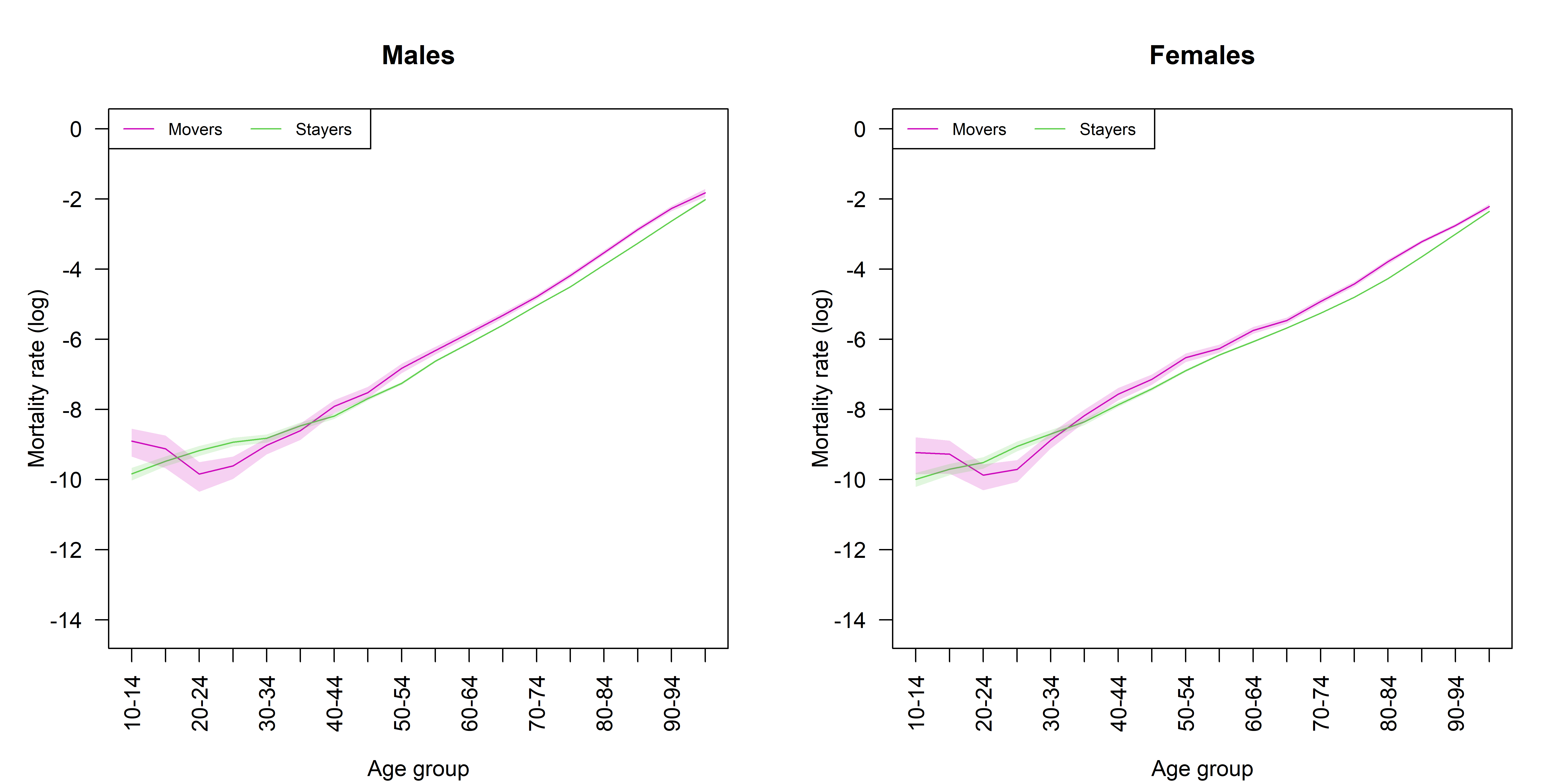


Data source: Statistics Netherlands. Note: Shaded areas indicate 95% confidence intervals. Some values could not be computed due to low numbers. The mortality rate (and its confidence interval) for females’ lifestyle-related mortality at age 15-19 of zero was approximated using linear interpolation.
